# Declines in social–emotional skills in college students during the COVID-19 pandemic

**DOI:** 10.3389/fpsyg.2024.1392058

**Published:** 2024-07-15

**Authors:** Janine Cerutti, Keith B. Burt, Robert W. Moeller, Martin Seehuus

**Affiliations:** ^1^Department of Psychological Science, University of Vermont, Burlington, VT, United States; ^2^Department of Psychology, Middlebury College, Middlebury, VT, United States; ^3^Vermont Psychological Services, Burlington, VT, United States

**Keywords:** college students, COVID-19, social–emotional, social–emotional skills, mental health

## Abstract

**Introduction:**

The present study investigated whether social–emotional skills in first year college students differed before and after the coronavirus disease (COVID-19) lockdowns.

**Methods:**

Participants (*N* = 1,685) consisted of first year college students (mean age 18.53 years) selected from a broader cohort enrolled in a longitudinal study on college mental health at liberal arts colleges in the United States. In a cohort-sequential design, participants completed an online survey assessing social–emotional skills in January of 2018, 2019, 2020, and 2022. Using analysis of covariance, we examined mean differences in social–emotional skills between students who were first years before (January 2018–2020) and after the lockdowns (January 2022), controlling for sociodemographic variables.

**Results:**

The post-lockdown group scored significantly lower on emotional control and expressivity and marginally higher on social sensitivity compared to the pre-lockdown group. No group differences in social/emotional expressivity or social control were detected.

**Discussion:**

These findings indicate that the COVID-19 lockdowns impaired some, but not all, social–emotional skills in first year college students. Addressing social–emotional skills in college may help to reduce the COVID-19 mental health burden.

## 1 Introduction

Many schools, from K-12 to college, shifted to remote instruction in March of 2020 in response to the rapid escalation of the coronavirus disease (COVID-19) pandemic. For most students, this shift was a dramatic change in their educational and social experiences. In the United States, the Centers for Disease Control and Prevention (CDC) strongly recommended social distancing, the avoidance of crowded indoor spaces, and the use of a mask, all of which added both logistical and subjective barriers to social contact (Centers for Disease Control and Prevention, 2020). Regarding educational outcomes, the shift to remote instruction at home has been shown to have a negative and long-lasting impact on higher education students’ critical thinking skills (Lv et al., 2022). In addition to no longer attending classes in person, many of the everyday social interactions students had with friends, peers, and teachers were reduced or eliminated. This led to less in-person contact with peers and an increased sense of emotional distance from social contacts (Dotson et al., 2022). Making up for the loss of in-person contact, use of digital media for socializing increased (Pandya and Lodha, 2021). Despite increasingly connecting with peers via social media, rates of loneliness significantly increased for college students during the COVID-19 pandemic (Labrague et al., 2021). Like most others, students experienced the COVID-19 lockdowns as physically and psychologically stressful (Birmingham et al., 2021; Camacho-Zuñiga et al., 2021).

For late high school and early college students, this change in socialization came at a critical time. Emerging adulthood is a period of important exploration (Arnett, 2014), identity development (Chickering and Reisser, 1993), and the growth of mature interpersonal relationships (Martin, 2000; Foubert and Urbanski, 2006). At the same time, development of key social–emotional skills continues (Wood et al., 2018; Bos et al., 2020). The ongoing development of the social and emotional skills of reading emotion in others and regulating one’s own social and emotional states to match social and contextual demands may have been hindered by the context shift from face-to-face to screen-mediated interactions (Isohätälä et al., 2021). Considering the importance of schools as a context for social–emotional development (Roeser et al., 2000; Eccles and Roeser, 2011), this impact may be both substantial and long-lasting (Center for Reinventing Public Education, 2021). In addition to direct social effects, the impaired development of social–emotional skills may have a deleterious effect on students’ mental health (Payton et al., 2000; Greenberg et al., 2017).

### 1.1 Social–emotional skills and mental health

Even in the decade prior to the COVID-19 pandemic, there was substantial concern about the high rates of psychological distress among college students. College student mental health centers (Gallagher, 2015) and the American College Health Association’s *National College Health Assessment* (American College Health Association, 2018) reported that rates of mental health challenges were already rising significantly well before the 2020 pandemic. Duffy et al. (2019) note that between 2007 and 2018, data from both the National College Health Assessment and the Healthy Minds Study indicated increased rates of depression and anxiety among college students. Eisenberg (2019) offers a series of possible explanations for the rise in mental health issues among college students including suggesting that an increase in social media leads to impaired sleep (Hysing et al., 2015), which in turn leads to increases in depression and anxiety (Eisenberg et al., 2018).

Thus, the pandemic and the lockdowns associated with it made things worse, with rates of depression, anxiety and stress all increasing (Jones et al., 2021). Adolescents’ experiences of loneliness also increased during the pandemic (Branje and Morris, 2021). Labrague et al. (2021) note that while loneliness was prevalent among college students, differences in how students experienced the pandemic were likely influenced by their use of coping behaviors and social supports. Other researchers have found that the COVID-19 pandemic and related social restrictions had a significant impact on the mental health of adolescents, particularly in terms of their access to social support and disruptions to their daily routines (Knowles et al., 2022). The mental health of young adults has been affected by the social isolation brought about by COVID-19, and early studies on this topic have highlighted the need to understand how the pandemic has affected the social–emotional skills that are crucial for helping individuals manage difficult circumstances. In addition to the direct effect of the loss of skills, this impairment is harmful to the mental health of emerging adults. For example, using the Trait Meta Mood scale of emotional intelligence (Salovey et al., 1995), a cross-sectional analysis of a subset of data from the present study found emotional attention, clarity, and repair all predicted college students’ depression, anxiety, and stress scores (Moeller et al., 2020).

### 1.2 The wide-ranging sequelae of social–emotional skills

Social and emotional skills are an integral component of Emotional Intelligence as initially theorized by Mayer and Salovey (1997) and subsequently revised by Mayer et al. (2004). This theory is structured around a four-component model of ability, including: perception of emotions, utilization of emotional information for cognition, understanding of emotions, and the ability to regulate and manage one’s emotions (Mayer et al., 2004).

The Riggio (1986, 2005) social skills inventory measures critical aspects of this theoretical model. Riggio’s social skills inventory assesses social–emotional skills within the domains of verbal-social and nonverbal-emotional (Riggio, 1986). Within each of the two domains, the measure captures three types of skills—encoding/expressivity skills, decoding/sensitivity skills and regulation/control skills (Riggio, 2005)—for a total of six constructs measured. The original 90-item measure (Riggio, 1986) has since been successfully abridged and tested with fewer items (Riggio and Carney, 2003; Segrin et al., 2007; Umphrey and Sherblom, 2014). Moeller and Seehuus (2019b) used this abridged inventory to determine that the social skills of expressivity (i.e., encoding communication), sensitivity (i.e., decoding communication), and control (i.e., regulating behaviors during communication) individually predict loneliness, depression, and anxiety in college students. Similarly, the social skills inventory has been found to be significantly associated with positive relations with others and overall psychological well-being (Suresh and Sandhu, 2012). Emotional control, the ability to recognize and regulate one’s social–emotional responses, has been found to be negatively associated with a sense of being a burden, thwarted belongingness, and suicidal ideation (Umphrey et al., 2021).

Emotional sensitivity, social expressivity and social control have all been documented to be associated with undergraduates’ reported levels of happiness, while social expressivity and social control were associated with self-esteem (Panchal and Joshi, 2013). In another study of university students, the social skills inventory was negatively correlated with loneliness and positively correlated with life satisfaction (Ozben, 2013). Umphrey and Sherblom (2014) found that the social skills inventory was positively correlated with hope, self-compassion, and life satisfaction.

Additionally, social–emotional skills are important for academic performance. Iqbal et al. (2021) note that emotional intelligence is an important factor in predicting academic performance, specifically in the context of the COVID-19 pandemic. Others including Koc and Turan (2018) note all six of the subscales of the social skills inventory were associated with college students’ cultural intelligence. This is particularly important as part of students’ ability to navigate the social world of colleges is to engage in the multicultural dynamics of a college.

### 1.3 The present study

Given the critical role that social–emotional skills play in the lives of emerging adults, the question of the impact of the COVID-19 lockdowns on the development of social–emotional skills looms large. Mayer and Salovey (1997)’s Emotional Intelligence theory provides a framework for how emotional capabilities and cognition underpin social–emotional skills, highlighting how understanding and managing emotions enhance everyday social interactions. Research has consistently shown that cognitive and affective processes are deeply intertwined, such that cognitive processes can up- or down-regulate emotional states and vice versa (Campos et al., 2004; Ochsner and Gross, 2005; Kray et al., 2020). Cognitive and affective networks function together to enhance the use of social–emotional skills in everyday life through attentional control, cognitive flexibility, emotion regulation, and empathy (Beauchamp and Anderson, 2010). Among young adults, the COVID-19 pandemic lockdowns and resulting social isolation have negatively impacted cognitive domains, including executive functioning, and affective domains, including emotion recognition and decreased positive emotions (Carbone et al., 2021; Bland et al., 2022; Panteli et al., 2022; Murtaza et al., 2023).

The COVID-19 pandemic lockdowns’ impact on cognitive and affective processes has important implications for the development and refinement of social–emotional skills. Based on Emotional Intelligence Theory (Mayer and Salovey, 1997), heightened stress, uncertainty, and anxiety during the COVID-19 pandemic lockdowns (Varma et al., 2021) may have overwhelmed young adults’ ability to manage emotions effectively, leading to maladaptive emotion regulation strategies and unhelpful social interactions. Evidence also suggests that foundational components of social–emotional skills, such as emotion recognition (Carbon and Serrano, 2021; Pazhoohi et al., 2021; Grahlow et al., 2022), assessment of emotional intensity (Tsantani et al., 2022), and emotional mimicry (Kastendieck et al., 2022), were all impaired by the facial coverings common during the pandemic. Moreover, Bandura’s Social Cognitive Theory posits that individuals’ social–emotional skills develop through observing, modeling, and reinforcing behaviors in social interactions (Bandura, 1997; Glanz et al., 2015). Social distancing and remote learning during the pandemic may have further hindered the development and refinement of these skills through limited face-to-face interactions and decreased social interactions (Wu et al., 2022), leading to impaired mental health specifically among college students (Thompson et al., 2021; Olson et al., 2023). Collectively, these theoretical perspectives suggest that the COVID-19 pandemic lockdowns may be associated with impairment in social–emotional skills through multiple mechanisms, including disruptions in emotional and cognitive abilities and social learning opportunities.

However, it remains unclear how social–emotional skills were specifically impacted by the COVID-19 pandemic lockdowns. To identify the effect of COVID-19 on the development of social–emotional skills in college students, we compared pre- and post-lockdown measurements of social–emotional skills in first year college students from two liberal arts colleges using data from a five-year longitudinal study of college student mental health spanning from 2018 to 2022. We focused on first year students for several reasons. First, by focusing on first year students we can assess their incoming social–emotional skills and mental health before their college experiences have (potentially) altered their psychosocial skills. Second, the transition to college occurs during the transition from late adolescence to emerging adulthood, a key developmental period in psychosocial adjustment (Schulenberg et al., 2004). Thus, an assessment of first year students would capture the disruption in the normal developmental trajectory more clearly than if older students were included. Finally, these compound, complex, and simultaneous adjustments—transitioning physically, emotionally, and intellectually a college environment—are notoriously difficult and associated with a significant dropout rate (Schneider, 2010; Aulck et al., 2019). By limiting our analysis to first year students, we are thus more likely to capture the breadth of college student experience. Further, we excluded data collected in January 2021 from analysis. In January 2021, the COVID-19 lockdown was in effect for some students and not others; thus, a sample collected in January of 2021 would have been drawn from a qualitatively distinct population, limiting interpretability.

Based on Emotional Intelligence Theory and Social Cognitive Theory, we hypothesized that the COVID-19-related lockdowns would be associated with impaired social–emotional skills across all six social and emotional skill subdomains analyzed, and that this effect would be evident when comparing first year students post-lockdown (January 2022) to first year students pre-lockdown (January 2018–January 2020).

## 2 Materials and methods

### 2.1 Procedure

The study procedure received approval from the Institutional Review Board (IRB) at the corresponding author’s institution. The current study is a secondary analysis of a cross-sequential study exploring psychosocial skills and mental health among undergraduate college students at two liberal arts colleges located in the Northeast United States. Data collection for the parent study occurred in five separate waves during January of 2018, 2019, 2020, 2021, and 2022. At each wave, all currently enrolled undergraduate students at both colleges (ranging from *N* = 4,348 to 4,679) received an email inviting students to participate in a study seeking to explore how students’ experiences with stress and other mental health factors change over time. The email contained a unique link to an online Qualtrics survey where students completed study measures. Students who consented and completed the survey were offered the chance to enter a raffle to win a $50 or $100 Amazon gift card. Each participant could complete a survey every year. Across the five waves, *N* = 6,348 unique students provided a total of 8,966 responses, a total study response rate across 5 years of data collection of 40%.

### 2.2 Participant characteristics

Of the 8,966 survey responses, surveys not from first year students and those that did not respond to the question of what year in college they were (*n* = 6,597) were excluded. Participants (*n* = 48) were then dropped if their responses did not pass one or more data quality test (e.g., participants were excluded if they had straight line response pattern across more than one measure, or inconsistent data within a single data collection wave), and/or if they did not report age or reported an age equal to or beyond 28 years, the known highest value for this population. We further removed responses (*n* = 157) who did not complete all 24 survey questions from the abridged version of the Social Skills Inventory (SSI) (Riggio, 1986), our primary variable of interest. Preliminary analyses determined there were no significant differences between participants who completed and did not complete SSI questions on age, gender, race, and socioeconomic status. Finally, as described above, participants from the January 2021 wave (*n* = 479) were excluded from the current study, resulting in a total analytic sample of 1,685 participants. Among the final analytic sample (*N* = 1,685), there were 533 participants in 2018 (31.6%), 551 in 2019 (32.7%), 174 in 2020 (10.3%), and 427 in 2022 (25.3%) waves.

To determine whether SSI scores differed before and after the COVID-19 lockdowns, we collapsed waves 2018–2020 to create a pre-lockdown group (*n* = 1,258). Pairwise comparisons were run to identify any significant differences in demographics or SSI measures between 2018, 2019, and 2020 waves, with no significant differences found. The post-lockdown group (*n* = 427) included participants from 2022. Of note, data collection occurred in January of each academic year and thus 2020 data was collected prior to the lockdowns beginning in March 2020.

### 2.3 Measures

#### 2.3.1 Sociodemographics

During the online survey, participants were asked to self-report their age, gender, race, and perceived socioeconomic status. Age was modeled as a continuous variable, while gender, race, and socioeconomic status were dummy-coded.

#### 2.3.2 Social–emotional skills

Social–emotional skills were measured via an abridged version of the 90 Social Skills Inventory (SSI) (Riggio, 1986). The abridged version of the SSI, which has been validated in prior work (Oldmeadow et al., 2013), includes a total of 24 questions capturing two domains of social ability: emotional (related to non-verbal skills) and social (related to verbal skills). The emotional and social domains are each broken down into the following three subdomains:

1. Expressivity: emotional expressivity (EE) and social expressivity (SE)
2. Sensitivity: emotional sensitivity (ES) and social sensitivity (SS)
3. Control: emotional control (EC) and social control (SC)

Each subdomain assesses non-verbal (emotional) and verbal (social) aspects of social ability, respectively, and yields a total of six social skills measures (SSI measures). Each SSI measure includes four items on a five-point scale ranging from “Not at all like me” (1) to “Exactly like me” (5). Expressivity focuses on one’s ability to communicate non-verbally (EE; e.g., “I rarely show my feelings or emotions”) and verbally (SE; e.g., “At parties I enjoy talking to a lot of different people”). The sensitivity subdomain captures one’s ability to mediate communication non-verbally (ES; e.g., “I always seem to know what peoples’ true feelings are no matter how hard they try to conceal them”) and verbally (SS; e.g., “It is very important that other people like me”). Lastly, the control subdomain measures the ability to control and regulate behaviors non-verbally (EC; e.g., “I am rarely able to hide a strong emotion”) and verbally (SC; e.g., “I am usually very good at leading group discussion”). Higher scores on all SSI measures, excluding SS, indicate more adaptive social–emotional abilities. Higher scores on SS reflect poorer adaptation, as higher scores may signal hypervigilance regarding how others perceive oneself and sensitivity to criticism.

### 2.4 Analysis

Data were examined for normality using skewness and kurtosis, and Levene’s tests were used to test the homogeneity of variance assumption. Descriptive statistics were generated for sociodemographic variables, and we compared differences in sociodemographics between pre- and post-lockdown groups using t-tests and chi-square tests for continuous and categorical variables, respectively. We conducted a total of six one-way analyses of covariance (ANCOVAs) to assess whether mean scores of SSI measures were significantly different between the pre-lockdown and post-lockdown groups, controlling for gender, race, and socioeconomic status. We included these variables as covariates given prior research showing that social and emotional skills vary by gender, race, and socioeconomic status (de Ridder et al., 2012; Kuo et al., 2020; Gruijters et al., 2024). To estimate the SSI measures’ internal consistencies, Cronbach’s alpha coefficients were generated for pre- and post-lockdown groups. We further performed sensitivity analyses to examine if any potentially significant results related to emotional expressivity (EE) were driven by items related to concerns of touching others following the wake of COVID-19. Specifically, EE contained two items related to touch, for example “I usually feel uncomfortable touching other people.” We created a sum score of the two EE items that were unrelated to touch (i.e., “Sometimes I have trouble making my friends and family realize how angry or upset I am with them,” and “I rarely show my feelings or emotions”). As a robustness check, we ran an ANCOVA, controlling for gender, race, and socioeconomic status, to examine if there was a significant difference on this two-item EE sum score between the pre- and post-lockdown groups. All analyses were performed in R version 3.6 (R Core Team, 2022).

## 3 Results

Among the final analytic sample (*N* = 1,685), there was no missing data on age, gender, or socioeconomic status; however, 86 observations (5.10% of the sample) were missing on race. Demographic details are shown in Table 1. In the full analytic sample, the mean age of first year students was 18.53 years, approximately 56.8% of the sample identified as female, and 69.5% identified as white/non-Hispanic. There were significant differences in the distributions of gender, race, and socioeconomic status between pre- and post-lockdown groups (see Table 1). Age was not significantly different between pre- and post-lockdown groups and was therefore removed as a covariate in the ANCOVAs.

**Table 1:** Demographics of full sample and pre-/post-COVID lockdown groups.

	Full sample (*N* = 1,685)	Pre-lockdown (*n =* 1,258)	Post-lockdown (*n* = 427)	*p*-value^a^	Missing (%)
Age *Mean (SD)*	18.53 (0.65)	18.52 (0.64)	18.57 (0.68)	.185	0
Gender (%)				<.001	0
Man	685 (40.7%)	520 (41.3%)	165 (38.6%)		
Woman	957 (56.8%)	721 (57.3%)	236 (55.3%)		
TGD	39 (2.3%)	15 (1.2%)	24 (5.6%)		
Prefer not to answer	4 (0.2%)	2 (0.2%)	2 (0.5%)		
Race (%)				.031	5.1
Native American	6 (0.4%)	3 (0.3%)	3 (0.8%)		
Asian	199 (12.4%)	143 (11.9%)	56 (14.0%)		
Black	114 (7.1%)	75 (6.3%)	39 (9.8%)		
Hawaiian or Pacific Islander	3 (0.2%)	3 (0.3%)	0 (0%)		
White	1,112 (69.5%)	857 (71.5%)	255 (63.7%)		
More than one	121 (7.6%)	84 (7.0%)	37 (9.2%)		
Prefer not to answer	44 (2.8%)	34 (2.8%)	10 (2.5%)		
SES (%)				.008	0
Lower	231 (13.7%)	155 (12.3%)	76 (17.8%)		
Middle	888 (52.7%)	684 (54.4%)	204 (47.8%)		
Upper	566 (33.6%)	419 (33.3%)	147 (34.4%)		

^a^Comparison between pre- and post-COVID lockdown groups. SES, socioeconomic status; TGD, transgender and gender-diverse. *p*-values assessed whether distributions of covariates were different between pre- and post-COVID lockdown groups. *p*-values were determined from *t*-tests for continuous variables and chi-square tests for categorical variables.

The Cronbach’s alphas for emotional sensitivity (ES), emotional control (EC), social expressivity (SE), social sensitivity (SS), and social control (SC) were found to be highly reliable in both pre- and post-lockdown groups (4 items each; Cronbach’s α ranged from .78–.92). However, Cronbach’s alpha coefficients for the 4-item emotional expressivity (EE) subdomain in the pre-lockdown and post-lockdown group were .57 and .60, respectively, suggesting a lower level of reliability for the EE. We found that social–emotional skill scores were approximately normally distributed with skewness and kurtosis values within +/− 1.00 for both pre- and post-lockdown groups. Levene’s tests indicated no significant differences (all *p*s > .05) in the variance of social–emotional skills between pre- and post-lockdown groups, suggesting the assumption of homogeneity of variance was met.

ANCOVAs were conducted on the dataset using complete cases, which involved the removal of 86 observations with missing data on race. As a result, the final sample size for the ANCOVAs consisted of 1,599 participants (pre-lockdown group: *n =* 1,199; post-lockdown group: *n* = 400). Results from the six ANCOVAs are presented in Tables 2, 3 for emotional-focused (nonverbal) and social-focused (verbal) skill subdomains, respectively. All ANCOVAs controlled for the following covariates: gender, race, and socioeconomic status. Our hypothesis was partially supported: mean SSI scores significantly differed between pre- and post-lockdown groups in two out of six subdomains. Controlling for covariates, the post-lockdown group (*M* = 12.49, *SD* = 3.13) had significantly lower scores on EE than the pre-lockdown group (*M* = 12.97, *SD* = 3.03), *F*(1, 1,586) = 5.30, *p* = .021, η^2^ = .003, suggesting a poorer ability to communicate emotions non-verbally after COVID-19 lockdowns. Additionally, the post-lockdown group (*M* = 13.3, *SD* = 3.65) scored significantly lower on EC than the pre-lockdown group (*M* = 14.48, *SD* = 3.44), *F*(1, 1,586) = 5.71, *p* = .017, η^2^ = .004, suggesting that, after the COVID-19 lockdown, students were less able to control and regulate their emotions than before the lockdowns. Lastly, the post-lockdown group (*M* = 14.38, *SD* = 3.63) had higher scores on SS than the pre-lockdown group (*M* = 13.95, *SD* = 3.70), *F*(1, 1,586) = 3.76, *p* = .052, η^2^ = .002, although this difference was only marginally significant. Given that higher scores on this subscale indicate more sensitivity to criticism and concern regarding how one is perceived by others, this result suggests poorer adaptation following the COVID-19 lockdowns. There were no significant differences between pre- and post-lockdown groups on ES, SE, or SC measures.

**Table 2:** ANCOVAs comparing pre- and post-COVID lockdown groups on emotional skill subdomains (*N* = 1,599).

	Pre-lockdown (*n* = 1,199)		Post-lockdown (*n* = 400)				
	*M*	*SD*	*M*	*SD*	*F*	*p*-value	η^2^
Emotional Expressivity*	12.97	3.03	12.49	3.13	5.30	.021	.003
Covariates							
Gender**					4.82	.002	.009
Race					1.18	.313	.004
SES					1.02	.360	.001
Emotional sensitivity	13.02	3.38	13.18	3.40	1.11	.292	.001
Covariates							
Gender***					5.74	.001	.011
Race					1.00	.422	.004
SES					0.51	.604	.001
Emotional Control**	14.48	3.44	13.93	3.65	5.71	.017	.004
Covariates							
Gender***					15.41	< .001	.028
Race					1.16	.323	.004
SES					0.17	.843	< .001

*p < .05; **p < .01; ***p < .001. SES, socioeconomic status. Three ANCOVAs between pre- and post-COVID lockdown groups are presented for three emotional subdomains (emotional expressivity, emotional sensitivity, and emotional control), controlling for gender, race, and SES. Covariate results are presented as omnibus *F*-tests for sets of dummy-coded variables entered at each step of the model.

**Table 3:** ANCOVAs comparing pre- and post-COVID lockdown groups on social skill subdomains (*N* = 1,599).

	Pre-lockdown (*n* = 1,199)		Post-lockdown (*n* = 400)				
	*M*	*SD*	*M*	*SD*	*F*	*p*-value	η^2^
Social Expressivity	12.46	4.36	12.06	4.42	1.40	.236	.001
Covariates							
Gender					1.56	.198	.003
Race*					2.22	.039	.008
SES**					6.86	.001	.008
Social Sensitivity	13.95	3.70	14.38	3.63	3.76	.052	.002
Covariates							
Gender***					19.37	< .001	.034
Race***					4.60	< .001	.016
SES*					4.46	.012	.005
Social Control	12.23	3.51	12.09	3.50	0.05	.829	<.001
Covariates							
Gender*					3.60	.013	.007
Race**					3.55	.002	.013
SES**					5.11	.006	.006

*p < .05; **p < .01; ***p < .001. SES, socioeconomic status. Three ANCOVAs between pre- and post-COVID lockdown groups are presented for three social subdomains (social expressivity; social sensitivity; social control), controlling for gender, race, and SES. Covariate results are presented as omnibus F-tests for sets of dummy-coded variables entered at each step of the model.

Sensitivity analysis revealed that both of the two EE items unrelated to touch were significantly, positively correlated with the original EE measure (*r* = .59, *p* < .01; *r* = .69, *p* < .01). After controlling for covariates, the difference between the pre-lockdown group (*M* = 6.99, *SD* = 1.80) and the post-lockdown group (*M* = 6.74, *SD* = 1.88) on the summed two-item EE variable unrelated to touch was marginally significant, *F*(1, 1,586) = 3.66, *p* = .056.

The main effects of covariates on emotional and social skill subdomains are presented in Tables 4, 5, respectively. Only gender was significantly related to all three emotional skill subdomains. For social skill subdomains, all covariates were significantly related to SS and SC. Lastly, only race and socioeconomic status were significantly related to SE.

**Table 4:** Main effects of covariates on emotional skill subdomains (*N* = 1,599).

	EE			ES			EC		
	*M* (*SD*)	*t*	*p-*value	*M* (*SD*)	*t*	*p-*value	*M* (*SD*)	*t*	*p-*value
Gender									
Woman^a^	13.07 (3.15)	–	–	13.31 (3.28)	–	–	13.90 (3.63)	–	–
Man	**12.62 (2.88)**	**−3.02**	**.021**	12.79 (3.42)	**−3.08**	**.002**	**15.06 (3.17)**	**6.37**	**<.001**
TGD	**11.58 (3.57)**	**−2.67**	**.003**	11.71 (4.29)	**−3.14**	**.002**	12.92 (3.77)	−1.45	.146
Prefer not to answer	12.75 (2.87)	0.01	.991	13.50 (5.74)	0.01	.993	13.25 (3.10)	−0.47	.640
Race									
White^a^	12.94 (3.06)	–	–	13.01 (3.31)	–	–	14.39 (3.55)	–	–
Native American	13.83 (2.64)	0.71	.479	13.17 (2.79)	−0.13	.895	12.00 (5.51)	−1.34	.181
Asian	12.66 (2.84)	−1.00	.239	13.02 (3.57)	−0.05	.961	13.81 (3.54)	−1.72	.086
Black	12.54 (3.28)	−0.89	.372	12.89 (3.65)	−0.39	.696	14.52 (3.05)	0.52	.605
Hawaiian or Pacific Islander	16.00 (4.00)	1.91	.056	14.33 (4.04)	0.95	.342	14.00 (0.00)	−0.09	.926
More than one	12.74 (3.18)	−0.37	.712	**13.65 (3.26)**	**2.06**	**.040**	14.60 (3.33)	0.64	.524
Prefer not to answer	12.27 (3.26)	−1.15	.251	13.34 (3.96)	0.73	.467	14.75 (3.19)	0.87	.384
SES									
Upper^a^	13.03 (2.93)	–	–	13.16 (3.30)	–	–	14.41 (3.50)	–	–
Middle	12.79 (3.15)	−1.38	.168	12.98 (3.40)	−0.97	.331	14.29 (3.56)	−0.23	.819
Lower	12.63 (3.04)	−0.91	.361	13.14 (3.58)	−0.17	.868	14.40 (3.19)	0.40	.693

^a^ Reference group. EC, emotional control; EE, emotional expressivity; ES, emotional sensitivity; SES, socioeconomic status; TGD, transgender and gender-diverse. The main effects of gender, race, and socioeconomic status from the ANCOVAs are presented for emotional skill subdomains: emotional expressivity, emotional sensitivity, and emotional control. Bolding indicates significant effects with *p*-values under .05.

**Table 5:** Main effects of covariates on social skill subdomains (*N* = 1,599).

	SE			SS			SC		
	*M* (*SD*)	*t*	*p-*value	*M* (*SD*)	*t*	*p-*value	*M* (*SD*)	*t*	*p-*value
Gender									
Woman^a^	12.40 (4.37)	–	–	14.50 (3.58)	–	–	12.04 (3.67)	–	–
Man	12.37 (3.69)	−0.58	.561	**13.31 (3.69)**	**−6.81**	**< .001**	**12.51 (3.18)**	**2.10**	**.036**
TGD	10.97 (3.64)	−1.81	.070	**15.95 (3.64)**	**2.49**	**.013**	**10.66 (4.31)**	**−2.24**	**.025**
Prefer not to answer	14.75 (5.32)	1.07	.287	14/50 (5.32)	0.36	.716	12.00 (2.71)	−0.04	.971
Race									
White^a^	12.58 (4.33)	–	–	14.28 (3.68)	–	–	12.45 (3.49)	–	–
Native American	10.33 (4.18)	−0.83	.405	**10.83 (2.64)**	**−2.32**	**.021**	10.50 (4.41)	−1.15	.248
Asian	**11.30 (4.48)**	**−3.03**	**.002**	14.18 (3.59)	−0.34	.734	**11.11 (3.23)**	**−4.43**	**< .001**
Black	11.99 (4.48)	−0.16	.873	**12.60 (3.34)**	**−3.86**	**< .001**	11.68 (3.28)	−1.58	.115
Hawaiian or Pacific Islander	15.67 (4.04)	1.45	.147	11.67 (2.52)	−1.40	.163	12.33 (3.21)	0.16	.873
More than one	12.66 (4.45)	0.61	.540	14.02 (3.59)	−0.61	.542	12.31 (3.77)	−0.33	.745
Prefer not to answer	12.34 (3.91)	0.24	.814	**12.41 (4.33)**	**−3.06**	**.002**	11.86 (3.78)	−0.63	.531
SES									
Upper^a^	12.96 (4.40)	–	–	14.46 (3.66)	–	–	12.71 (3.63)	–	–
Middle	**12.15 (4.36)**	**−3.11**	**.002**	**13.01 (3.92)**	**−1.97**	**.049**	**11.82 (3.35)**	**−3.20**	**.001**
Lower	**11.51 (4.16)**	**−3.12**	**.002**	**14.01 (3.61)**	**−2.86**	**.004**	11.94 (3.42)	−1.46	.144

^a^Reference group. SC, social control; SE, social expressivity; SS, social sensitivity; SES, socioeconomic status; TGD, transgender and gender-diverse. The main effects of gender, race, and socioeconomic status from the ANCOVAs are presented for social skill subdomains: social expressivity, social sensitivity, and social control. Bolding indicates significant effects with *p*-values under .05.

## 4 Discussion

Social–emotional skills play an important role in students’ academic achievement, mental health, and other life outcomes (Greenberg et al., 2017; Alzahrani et al., 2019). The current study investigated whether social–emotional skills differed between first year college students in the United States before and after the lockdowns related to the COVID-19 pandemic. To our knowledge, this study is the first to assess the impact of the COVID-19 lockdowns on the development of social–emotional skills in college students. Our hypothesis that students’ social–emotional skills would decline following COVID-19 lockdowns was partially supported. The COVID-19 pandemic lockdowns had a negative impact on some of the social and emotional skills of incoming college students, but other domains were unaffected. Compared to pre-pandemic first year college students, first year college students assessed in 2022 had lower emotional control and expressivity skills, and marginally higher social sensitivity skills, after controlling for gender, race, and socioeconomic status. That suggests that post-lockdown, students were coming out of high school with more poorly developed skills to communicate emotions non-verbally (EE, emotional expressivity) and to control and regulate their emotional displays (EC, emotional control). Further, social sensitivity (SS), or the ability to navigate social norms, was higher. Higher SS scores are theorized to be associated with increased social anxiety and self-consciousness (Riggio, 1986). Differences were not observed in emotional sensitivity (ES), or the ability to perceive non-verbal affective communications, social expressivity (SE), which is the ability to communicate emotional states verbally, and social control (SC), which is the ability to manage self-presentation in social settings.

Although we hypothesized that all domains of social and emotional skills would be impaired, these results paint a more nuanced picture of the effect of the lockdowns. The results describe a cohort reaching for more emotional connection while self-consciously aware of their own impaired social skills; a cohort that lacks the real-world experience that could reduce the intensity of that self-consciousness with repeated exposures.

The pandemic lockdowns were associated with a reduction in at least the variety if not quantity of in-person interactions. We speculate that it was that change that led to the observed reduction in non-verbal communication skills, measured by a lower EE score. Further, we suggest that the stress and emotional intensity of the pandemic, while paired with a reduction in access to the adaptive social coping opportunities normally present, has left a cohort with reduced abilities to manage their own emotional displays (whether through lack of practice or because a video-mediated communication alters the normal non-verbal communication methods; see Schaarschmidt and Koehler (2021) for discussion) and hyperaware of their own social presentation. These data suggest that lockdowns did not impair attention and sympathy toward the emotional states of others, and neither did they alter verbal communication or social presentation skills.

There are important limitations to these results. Most notably, students from small liberal arts colleges have important differences when compared to other college students, or to emerging adults who did not pursue college, including differences in race, ethnicity, educational attainment, and socioeconomic status (Blanco et al., 2008; Han et al., 2016; Baldwin et al., 2017). Collectively these factors may limit the generalizability of our findings. These results are unable to speak to how those differences may have an effect on these results or conclusions, and caution is warranted in generalizing these conclusions. Further, although self-report is the most common way of assessing social–emotional skills, all self-report methods have inherent self-presentation limitations that should be considered (Althubaiti, 2016). There may have been a selection bias, which influenced which students chose to participate in the study and those students who did not. It is possible that our sample included students who had more interest in mental health subject matter, or had higher levels of social–emotional skills. Additionally, while our analytic approach allowed us to address an emerging and time-sensitive question on changes in social–emotional skills associated with the COVID-19 pandemic lockdowns, our results may be subject to measurement error. Future research should test more complex models, such as those incorporating latent variables to account for measurement error, to further investigate the trajectories of social–emotional skills throughout the COVID-19 pandemic.

Lastly, the COVID-19 lockdowns were not experienced uniformly across the United States. Although the significant effects detected were robust to key sociodemographic covariates, other unanticipated and thus unmeasured factors likely influenced the effect of the lockdowns on students’ social–emotional skills. Future research is warranted to explore how factors such as home life, alternative remote learning methods, and the severity of state-level lockdown restrictions impacted students’ social–emotional skills development.

This is not the only interpretation of this pattern of results, and further work will be necessary to confirm our speculative causal mechanism and compare its strength with other factors. There is some urgency to understanding this process because these findings suggest that the COVID-19 associated lockdowns had deleterious effects on the social–emotional skills of incoming college students. Given the mental health implications of social–emotional skills, particularly amongst college students (Payton et al., 2000; Greenberg et al., 2017), these changes may be part of the mechanism connecting the lockdowns to the observed decline in college student mental health (Conrad et al., 2021; Copeland et al., 2021). If so, they are potentially a target for intervention. Combined with the evidence that the mental health of college students has been declining steadily over the past decade (Kruisselbrink Flatt, 2013; Pedrelli et al., 2015; Moeller and Seehuus, 2019b), these results both suggest that, absent some kind of intervention, the emotional impact of the lockdowns is likely to further accelerate the trend toward increased psychological distress amongst college students.

Although social skills are widely assessed and implemented, particularly in primary school settings (la Greca and Santogrossi, 1980; Ogilvy, 1994), that is less true for the general college population (Brown, 2013; Moeller and Seehuus, 2019a; Kul and Gönültaş, 2022). It is likely that explicitly addressing social–emotional skills amongst rising and current college students would be of benefit in a number of domains; given the impact of the pandemic lockdowns on those social–emotional skills, teaching these social and emotional skills may be an important step in mitigating the impact of the lockdowns. Future research should explore such interventions.

## Data availability statement

The data analyzed in this study is subject to the following licenses/restrictions: the data that support the findings of this study will be made available in the National Data Archive upon publication. Requests to access these datasets should be directed to RM, the corresponding author at rmoeller@middlebury.edu.

## Ethics statement

The studies involving humans were approved by Institutional Review Board, Middlebury College. The studies were conducted in accordance with the local legislation and institutional requirements. The participants provided their written informed consent to participate in this study.

## Author contributions

JC: Writing – review & editing, Writing – original draft, Methodology, Formal analysis, Conceptualization. KB: Writing – review & editing, Writing – original draft, Methodology, Formal analysis, Conceptualization. RM: Writing – review & editing, Writing – original draft, Methodology, Funding acquisition, Data curation, Conceptualization. MS: Formal analysis, Writing – review & editing, Writing – original draft, Methodology, Data curation, Conceptualization.
